# Sex, BMI, skeletal maturation, and height among children and adolescents in high-altitude Xizang: a cross-sectional study

**DOI:** 10.3389/fendo.2026.1942630

**Published:** 2026-10-06

**Authors:** Lihui Yang, Xiaoyong Yuan, Xiaolong Wu, Shuyou Meng, Xuemei Lv, Zhining Zhang

**Affiliations:** 1 Department of Science and Education, People’s Hospital of Xizang Autonomous Region, Lhasa, Xizang, China; 2 Department of Endocrinology, Peking University First Hospital, Beijing, China; 3 School of Medicine, Tibet University, Lhasa, Xizang, China; 4 Department of Endocrinology, People’s Hospital of Xizang Autonomous Region, Lhasa, Xizang, China; 5 Graduate School of Medicine, Kyoto University, Kyoto, Japan

**Keywords:** body mass index, bone age, children and adolescents, high altitude, linear growth, pubertal development, skeletal maturation, Xizang

## Abstract

**Background:**

Skeletal maturation influences the interpretation of childhood height, but its relationship with sex, body mass index (BMI), and attained height in children living at high altitude remains incompletely characterized. We examined concurrent associations among sex, BMI, bone-age deviation, and height relative to national reference values in children and adolescents living in Lhasa, Xizang.

**Methods:**

This cross-sectional study included 199 participants aged 3–16 years (101 girls and 98 boys). Bone-age deviation was defined as bone age minus chronological age. Height difference and BMI difference were calculated relative to age- and sex-specific national reference values. Multivariable linear regression incorporated centered chronological age and its quadratic term, ethnicity, BMI difference, and pubertal status, with HC3 heteroscedasticity-consistent standard errors. Statistical indirect associations through bone-age deviation were estimated using 5,000 participant-level bootstrap resamples.

**Results:**

Mean bone-age deviation was −0.64 ± 1.14 years. After adjustment, boys had a 0.65-year lower bone-age deviation than girls (95% CI, −0.96 to −0.33; P < 0.001). Each 1-kg/m² increase in BMI difference was associated with a 0.06-year increase in bone-age deviation (95% CI, 0.01 to 0.10; P = 0.010). Each 1-year increase in bone-age deviation was associated with a 2.76-cm greater height difference from the national reference (95% CI, 1.82 to 3.70; P < 0.001). The sex-related statistical indirect association through bone-age deviation was −1.78 cm (percentile 95% CI, −2.89 to −0.83; BCa 95% CI, −3.02 to −0.90). The corresponding direct association had confidence intervals spanning zero. Principal findings were generally retained in sensitivity analyses using grouped Tanner stages and after exclusion of influential or extreme observations.

**Conclusion:**

In this Lhasa sample, boys showed greater skeletal maturation delay than girls, and skeletal maturity was strongly associated with current height relative to national references. Higher BMI relative to the corresponding reference was associated with more advanced skeletal maturation. These cross-sectional findings describe statistical associations and do not establish temporal mediation or an altitude-specific causal effect.

## Introduction

Assessment of childhood growth requires consideration of both attained height and biological maturity. Children of the same chronological age may differ substantially in the timing of their pubertal growth spurt and skeletal development. Longitudinal evidence indicates that hand skeletal maturity is closely related to the stage of the adolescent growth spurt and remaining growth, providing information beyond chronological age alone (1). Age- and sex-specific Chinese growth references provide benchmarks for evaluating attained height (2). Interpreting height relative to these references alongside bone-age deviation, defined as bone age minus chronological age, may therefore help distinguish differences in current stature from differences in maturational timing.

Growth assessment in children living on the Tibetan Plateau also requires attention to the population and environmental context. Earlier research documented impaired linear growth and nutritional problems among Tibetan children living at high altitude (3). More recently, a multicenter study reported greater bone-age delay among children residing at higher altitudes (4). These observations motivate further investigation of skeletal maturation in high-altitude settings. However, differences across locations may reflect multiple correlated influences, including nutritional circumstances, socioeconomic conditions, and population background. National growth references provide a useful external benchmark, but comparisons with these references cannot isolate the contribution of altitude or substitute for a contemporaneous low-altitude comparison group.

Sex, pubertal development, and body composition are particularly relevant to the relationship between skeletal maturation and height. The differing timing of the adolescent growth spurt in boys and girls means that adjustment for chronological age alone may not fully account for developmental differences (1). In a longitudinal Chinese cohort, persistently higher childhood trajectories of body mass index (BMI) and other body composition indicators were associated with a greater likelihood of advanced bone age at puberty (5). These findings support examining BMI and skeletal maturation jointly while considering pubertal development. Nevertheless, BMI is an indirect measure of adiposity and incorporates height in its calculation, complicating the interpretation of associations between BMI and height.

Genetic background provides another important context for variation in growth. Large genome-wide association studies have established the polygenic architecture of height and BMI (6, 7). Height research has also demonstrated differences in the predictive performance of genetic variants across ancestry groups, underscoring the importance of population context (6). At the level of rare skeletal disorders, studies of Vietnamese, Estonian, and Ukrainian patients with osteogenesis imperfecta have identified pathogenic variants in *COL1A1* and *COL1A2*, which encode the chains of type I collagen, and documented variation in their mutational spectra and clinical manifestations (8–10). These studies illustrate the importance of collagen integrity for skeletal health, although their findings cannot be directly extrapolated to common variation in BMI or skeletal maturation in children without these disorders. Genetic ancestry and environmental differences therefore warrant consideration when interpreting growth against external population references.

Against this background, the joint relationships among sex, BMI, skeletal maturation, and height within a high-altitude clinical sample warrant further characterization. The present cross-sectional study examined children and adolescents aged 3–16 years living in Lhasa, Xizang. We assessed sex-related differences in bone-age deviation and height relative to age- and sex-specific national reference means, the association of BMI difference from the corresponding reference mean with bone-age deviation, and the association between bone-age deviation and height difference. Additional analyses considered pubertal development and the robustness of these associations. Exploratory analyses evaluated statistical indirect associations involving bone-age deviation in the relationships of sex and BMI difference with height difference. These analyses were intended to characterize concurrent associations within the study sample; establishing temporal mediation or an altitude-specific causal effect was beyond the scope of the design.

## Materials and methods

### Study design and participants

This cross-sectional study was conducted in Lhasa, Xizang Autonomous Region, China, through the Department of Endocrinology at People’s Hospital of Xizang Autonomous Region. Healthy children and adolescents aged 3–16 years were recruited by convenience sampling between August 2021 and May 2023. Eligibility was assessed through medical history taking.

Participants were excluded if they had a history of perinatal brain injury, were born small for gestational age, were born preterm, had hepatic or renal insufficiency, cardiovascular or pulmonary disease, or intellectual disability.

Participants were required to have complete data on sex, chronological age, ethnicity, height, weight, height difference from the national reference, BMI difference from the national reference, Tanner stage, and bone age. Of the 200 participants in the source dataset, one participant whose ethnicity was neither Tibetan nor Han was excluded because this category contained only one individual. The final analytical sample therefore comprised 199 participants, including 101 girls and 98 boys.

No contemporaneous low-altitude comparison group was included. Age- and sex-specific national growth references were used as external benchmarks for height and BMI rather than as a contemporaneous control population. Accordingly, comparisons with the national reference values were used to characterize relative growth status within the study population and were not interpreted as estimates of an altitude-specific effect.

### Anthropometric assessment and reference-based growth measures

Demographic and anthropometric information was recorded using a structured study case-report form. Height was measured by trained healthcare personnel using a stadiometer conforming to national measurement standards. Participants were measured barefoot in an upright position with the back against the stadiometer, and height was recorded to the nearest 0.1 cm. Body weight was recorded in kilograms, and waist circumference was recorded in centimeters. Body mass index (BMI) was calculated as weight in kilograms divided by height in meters squared (kg/m²).

Height difference was defined as measured height minus the corresponding age- and sex-specific national reference value and was expressed in centimeters:

Height difference = measured height − age- and sex-specific national reference height.
Negative values indicated height below the corresponding reference value, whereas positive values indicated height above the reference value.
BMI difference was similarly defined as measured BMI minus the corresponding age- and sex-specific national reference value and was expressed in kg/m²:
BMI difference = measured BMI − age- and sex-specific national reference BMI.

Height difference and BMI difference were analyzed in their original units rather than as standardized z scores. Accordingly, the coefficient for sex in models of height difference represents a comparison of boys and girls in their deviation from their respective sex-specific national reference values and does not represent a direct comparison of absolute measured height between boys and girls.

Weight status was summarized descriptively according to the categories recorded in the study dataset as overweight, obesity, or other. These categories were used for descriptive presentation only and were not used as the principal BMI exposure in the regression analyses.

### Pubertal assessment

Pubertal development was classified according to Tanner stages I–V using the sex-specific Tanner staging descriptions recorded in the study assessment form. Pubertal-development information was obtained during the study assessment, and the recorded Tanner stage was used for analysis.

All five Tanner stages were retained in descriptive analyses to characterize the distribution of pubertal development in girls and boys. For the primary multivariable analyses, pubertal status was categorized as Tanner stage I versus Tanner stages II–V. To assess the potential influence of this categorization, sensitivity analyses used three groups: Tanner stage I, Tanner stages II–III, and Tanner stages IV–V. Tanner stage V was combined with stage IV because only one girl and one boy were classified as Tanner stage V. Additional subgroup analyses were restricted to participants at Tanner stage I.

### Bone-age assessment

Radiographs of the non-dominant hand and wrist were obtained for bone-age assessment. The left hand was examined in most participants, whereas the right hand was used for left-handed participants. Skeletal maturity was evaluated using the Chinese RUS-CHN bone-age assessment system.

Bone-age assessment followed a two-step clinical radiographic reading procedure involving two radiologists. The initial bone-age assessment was performed by a junior radiologist, after which the radiograph and bone-age determination were reviewed and confirmed by a senior radiologist. Both radiologists were blinded to the participants’ clinical and anthropometric information at the time of image interpretation. Formal inter-reader and intra-reader reliability coefficients were not available for the present analysis.

Bone-age deviation was calculated as:

Bone-age deviation = bone age − chronological age.

Bone-age deviation was expressed in years. Negative values indicated skeletal maturation behind chronological age, whereas positive values indicated skeletal maturation ahead of chronological age. Bone-age deviation was retained as a continuous variable in the principal regression analyses.

For descriptive presentation, bone-age deviation was categorized as delayed (<−1 year), within the defined normal range (−1 to 1 year, inclusive), or advanced (>1 year). This categorization was used only for descriptive summaries; all principal regression analyses used bone-age deviation as a continuous measure.

### Laboratory measurements

Fasting blood samples were collected from an antecubital vein and centrifuged before laboratory analysis. Measurements were performed by experienced laboratory personnel at People’s Hospital of Xizang Autonomous Region.

The endocrine and biochemical markers included in the exploratory analyses were parathyroid hormone (PTH), 25-hydroxyvitamin D, calcium, inorganic phosphorus, and alkaline phosphatase (ALP). All biomarkers were analyzed as continuous variables in their recorded measurement units.

### Statistical analysis

Statistical analyses were performed using R version 4.6.1 (R Foundation for Statistical Computing, Vienna, Austria). Robust covariance estimation, bootstrap analyses, and graphical presentation were performed using the *sandwich*, *boot*, and *ggplot2* packages, respectively.

Continuous variables were summarized as means and standard deviations (SDs), and categorical variables as counts and percentages. In descriptive analyses stratified by sex and detailed Tanner stage, chronological age was additionally summarized using the median, 25th and 75th percentiles, and observed range. SDs were not calculated for strata containing a single participant.

Descriptive comparisons between girls and boys used two-sided Welch t tests for continuous variables. Categorical distributions were compared using Pearson chi-square tests without continuity correction or Fisher exact tests when expected cell counts were less than five. Categorical P values represented comparisons of the complete distributions between sexes.

BMI was calculated from measured weight and height, and pubertal-status variables were derived from the recorded Tanner stage. Analyses were based on complete records for the variables required by each model, without imputation. Extreme or potentially influential observations were retained in the primary analyses and examined separately in sensitivity analyses.

### Multivariable regression analysis

Ordinary least-squares linear regression was used to examine associations with bone-age deviation and height difference. Chronological age was mean-centered, and the square of centered age was included to accommodate nonlinear associations with age. The same age-centering constant was retained in subgroup and sensitivity analyses.

The primary bone-age-deviation model included sex, centered chronological age, centered age squared, ethnicity, BMI difference, and pubertal status (Tanner stage I vs. Tanner stages II–V). Height difference was initially modeled using the same explanatory variables. Bone-age deviation was subsequently added to the height-difference model to estimate associations with height conditional on skeletal maturation. Girls, Tibetan ethnicity, and Tanner stage I were used as the reference categories.

Regression results were reported as unstandardized coefficients (β) with 95% confidence intervals (CIs). Type 3 heteroscedasticity-consistent (HC3) standard errors were used for statistical inference in the principal regression models. Confidence intervals and two-sided coefficient tests were based on the t distribution with the corresponding residual degrees of freedom.

### Statistical indirect, direct, and total associations

The association between sex and height difference was decomposed into statistical indirect, direct, and total associations. The indirect association through bone-age deviation was estimated using the product-of-coefficients approach by multiplying the sex coefficient from the bone-age-deviation model by the bone-age-deviation coefficient from the corresponding height-difference model.

The direct association was defined as the sex coefficient from the height-difference model after inclusion of bone-age deviation, whereas the total association was defined as the sex coefficient from the model before inclusion of bone-age deviation.

Sex-related estimates were expressed in centimeters of height difference for boys relative to girls. Models were adjusted for centered chronological age, centered age squared, ethnicity, BMI difference, and pubertal status (Tanner stage I vs. Tanner stages II–V).

An analogous analysis was performed for BMI difference, with estimates expressed per 1-kg/m² increase in BMI difference. These models were adjusted for sex, centered chronological age, centered age squared, ethnicity, and pubertal status. Because BMI mathematically incorporates height and because the direct and indirect associations could operate in opposite directions, the BMI-related decomposition was prespecified for cautious interpretation and was treated as exploratory.

Percentile and bias-corrected and accelerated (BCa) 95% CIs were estimated using 5,000 nonparametric participant-level bootstrap resamples. The component regression models were refitted within each bootstrap resample. Resamples in which the required models were rank deficient were not used for interval estimation. No proportion mediated was reported.

Because sex, BMI, bone age, and height were assessed cross-sectionally, these decompositions were interpreted as statistical indirect associations rather than evidence of temporal ordering, developmental mechanisms, or causal mediation.

### Sensitivity and subgroup analyses

Several sensitivity analyses were performed to evaluate the robustness of the principal regression associations.

First, the binary Tanner-stage variable was replaced by grouped Tanner stages I, II–III, and IV–V. Second, the principal models were repeated without adjustment for BMI difference. Third, potentially influential observations were excluded. Influential observations were defined as those with Cook’s distance >4/N in either the primary bone-age-deviation model or the height-difference model after adjustment for bone-age deviation. For the primary analytical sample of 199 participants, the corresponding threshold was 4/199 = 0.0201. Fourth, observations below the 1st percentile or above the 99th percentile of bone-age deviation were excluded.

Subgroup analyses of the principal associations were performed among Tibetan participants, participants at Tanner stage I, and participants aged <10 years. Ethnicity was omitted from Tibetan-only models and pubertal status was omitted from Tanner stage I models because these variables were constant within the corresponding subgroups. Subgroup estimates were considered descriptive assessments of robustness and were not interpreted as formal tests of between-subgroup heterogeneity.

Sex-related statistical indirect, direct, and total associations were additionally examined using grouped Tanner-stage adjustment, after exclusion of influential observations, after exclusion of observations in the extreme tails of the bone-age-deviation distribution, and within the Tibetan-only, Tanner stage I, and age-defined subgroups. The corresponding decomposition among participants aged ≥10 years was not estimable under the specified modeling procedure because unit leverage prevented reliable estimation.

BMI-related statistical decompositions were repeated using grouped Tanner-stage adjustment and after both observation-exclusion procedures.

### Interaction analyses and age-related patterns

Potential modification of the principal associations by sex was examined using HC3 robust Wald F tests.

Sex-by-age interaction was evaluated by jointly testing the sex × centered age and sex × centered age squared terms, corresponding to a two-degree-of-freedom test. These models included sex, centered chronological age, centered age squared, ethnicity, BMI difference, and grouped Tanner stage (I, II–III, and IV–V). The height-difference interaction model additionally included bone-age deviation.

Sex-by-BMI-difference interaction was evaluated in the bone-age-deviation model using a one-degree-of-freedom robust Wald F test. This model included sex, centered chronological age, centered age squared, ethnicity, BMI difference, pubertal status (Tanner stage I vs. Tanner stages II–V), and the sex × BMI-difference interaction term.

Sex-specific cross-sectional age patterns in bone-age deviation were additionally described using a model containing sex, centered chronological age, centered age squared, sex interactions with both age terms, ethnicity, and BMI difference. Model-based means were averaged over the observed distributions of ethnicity and BMI difference in the analytical sample and plotted between the sex-specific 2.5th and 97.5th percentiles of chronological age. Pointwise 95% CIs were calculated using the HC3 covariance matrix.

These curves were used to describe cross-sectional age associations and were not interpreted as longitudinal growth trajectories.

### Exploratory biomarker analyses

Associations of PTH, 25-hydroxyvitamin D, calcium, inorganic phosphorus, and ALP with bone-age deviation and height difference were examined in exploratory multivariable regression models. The five biomarkers were entered simultaneously.

The bone-age-deviation model was additionally adjusted for sex, centered chronological age, centered age squared, ethnicity, BMI difference, and grouped Tanner stage (I, II–III, and IV–V). The height-difference model additionally included bone-age deviation.

Regression coefficients and 95% CIs were estimated using HC3 standard errors. Benjamini–Hochberg false discovery rate (FDR) correction was applied across the 10 biomarker–outcome tests. Both unadjusted P values and FDR-adjusted P values (q values) were reported. These biomarker analyses were considered exploratory and hypothesis-generating.

### Model diagnostics and statistical inference

Model adequacy was evaluated using residual-versus-fitted plots, normal quantile–quantile plots of standardized residuals, leverage statistics, Cook’s distance, and heteroscedasticity diagnostics. Heteroscedasticity was evaluated using a studentized Breusch–Pagan-type test. HC3 standard errors were used for statistical inference in the principal regression models regardless of the heteroscedasticity-test result.

Models were assessed for rank deficiency and unit leverage before statistical inference. Coefficient tests and descriptive comparisons were two-sided, whereas interaction tests used the upper-tail probability of the corresponding F distribution. A two-sided P value <0.05 was considered statistically significant for the principal regression analyses. For the exploratory biomarker analyses, an FDR-adjusted q value <0.05 was considered statistically significant.

### Ethics statement

The study project received approval from the Ethics Committee of People’s Hospital of Xizang Autonomous Region (approval no. ME-TBHP-2021-029). Written informed consent was obtained from the participants’ parents or legal guardians. The study was conducted in accordance with the principles of the Declaration of Helsinki.

## Results

### Participant characteristics and skeletal maturation

Of the 200 participants in the source dataset, one participant whose ethnicity was neither Tibetan nor Han was excluded, leaving 199 participants for analysis: 101 girls and 98 boys. Mean age was 7.35 ± 3.07 years, and 157 participants (78.9%) were Tibetan. Mean measured height was 120.29 ± 19.77 cm, mean height difference from the age- and sex-specific national reference was −1.21 ± 7.88 cm, and mean BMI difference was 1.02 ± 2.98 kg/m² (**Table 1**).

**Table 1:** Characteristics of the study population, overall and by sex.

Characteristic	Overall	Girls	Boys	P value
Part A. Continuous characteristics				
Age, years	7.35 (3.07)	7.43 (3.14)	7.27 (3.00)	0.701
Height, cm	120.29 (19.77)	121.77 (19.60)	118.78 (19.93)	0.287
Height difference from national reference, cm	-1.21 (7.88)	0.52 (7.71)	-2.98 (7.69)	0.002
Weight, kg	25.24 (10.78)	25.26 (10.64)	25.21 (10.98)	0.974
BMI, kg/m²	16.83 (3.09)	16.41 (2.85)	17.27 (3.26)	0.048
BMI difference from national reference, kg/m²	1.02 (2.98)	0.75 (2.70)	1.30 (3.23)	0.202
Waist circumference, cm	56.17 (9.34)	55.35 (9.03)	57.02 (9.62)	0.208
Bone age, years	6.72 (3.36)	7.10 (3.38)	6.32 (3.31)	0.100
Bone-age deviation, years	-0.64 (1.14)	-0.33 (1.07)	-0.95 (1.13)	<0.001
PTH, pg/mL	65.73 (27.00)	70.98 (25.47)	60.32 (27.59)	0.005
25-hydroxyvitamin D, ng/mL	19.15 (6.68)	18.59 (6.51)	19.72 (6.85)	0.238
Calcium, mmol/L	2.43 (0.22)	2.42 (0.18)	2.43 (0.25)	0.583
Phosphorus, mmol/L	1.64 (0.26)	1.64 (0.27)	1.64 (0.26)	0.994
ALP, U/L	265.02 (71.79)	260.29 (76.55)	269.89 (66.58)	0.346
Part B. Categorical characteristics.				
Ethnicity: Tibetan	157 (78.9%)	77 (76.2%)	80 (81.6%)	0.351
Ethnicity: Han	42 (21.1%)	24 (23.8%)	18 (18.4%)	
Tanner stage: I	138 (69.3%)	73 (72.3%)	65 (66.3%)	0.778
Tanner stage: II	27 (13.6%)	11 (10.9%)	16 (16.3%)	
Tanner stage: III	18 (9.0%)	10 (9.9%)	8 (8.2%)	
Tanner stage: IV	14 (7.0%)	6 (5.9%)	8 (8.2%)	
Tanner stage: V	2 (1.0%)	1 (1.0%)	1 (1.0%)	
Bone-age status: Delayed	61 (30.7%)	20 (19.8%)	41 (41.8%)	0.003
Bone-age status: Normal	128 (64.3%)	75 (74.3%)	53 (54.1%)	
Bone-age status: Advanced	10 (5.0%)	6 (5.9%)	4 (4.1%)	
Weight category: Other	135 (67.8%)	72 (71.3%)	63 (64.3%)	0.281
Weight category: Overweight	39 (19.6%)	20 (19.8%)	19 (19.4%)	
Weight category: Obesity	25 (12.6%)	9 (8.9%)	16 (16.3%)	

Data are mean (SD) for continuous variables and n (%) for categorical variables. Between-sex comparisons were descriptive and used two-sided Welch t tests, Pearson chi-square tests, or Fisher exact tests as appropriate. The analytical sample included 199 participants (101 girls and 98 boys). Height difference and BMI difference represent the observed value minus the corresponding age- and sex-specific national reference value. Bone-age deviation was calculated as bone age minus chronological age; negative values indicate delayed skeletal maturation relative to chronological age. Delayed bone age was defined as bone-age deviation <−1 year, normal bone age as −1 to 1 year inclusive, and advanced bone age as >1 year. BMI, body mass index; PTH, parathyroid hormone; ALP, alkaline phosphatase; SD, standard deviation. Categorical percentages use the corresponding overall or sex-specific nonmissing denominator; each categorical P value compares the complete distribution between sexes. Weight categories retain the classifications recorded in the source dataset; “Other” denotes participants not coded as overweight or obese. National reference values are benchmarks, not a contemporaneous low-altitude control group.

Mean bone-age deviation was −0.64 ± 1.14 years. Boys had a more negative bone-age deviation than girls (−0.95 ± 1.13 vs. −0.33 ± 1.07 years; P < 0.001), with the distributions shown in **Figure 1**. Delayed bone age was observed in 61 participants (30.7%), including 41 boys (41.8%) and 20 girls (19.8%). Bone age was within the defined normal range in 128 participants (64.3%) and advanced in 10 (5.0%). The distribution of bone-age categories differed between sexes (P = 0.003; **Table 1**).

**Figure 1:**
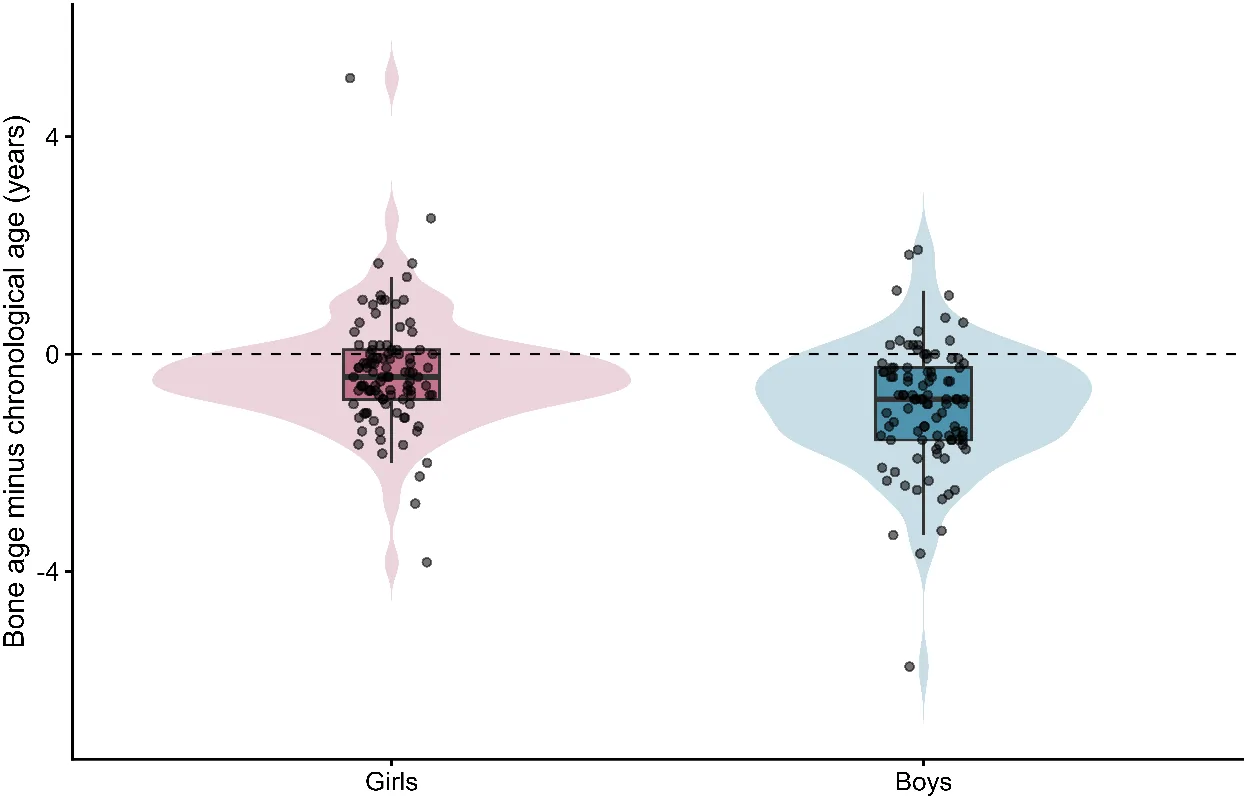
Distribution of bone-age deviation among girls and boys living in Xizang. Bone-age deviation is shown for 199 participants (101 girls and 98 boys). Pink and blue violins represent girls and boys, respectively, and show kernel density estimates. Boxes extend from the 25th to the 75th percentile, with the median indicated by the horizontal line; whiskers extend to the most extreme observations within 1.5 interquartile ranges of the box limits. Jittered points represent individual participants. Bone-age deviation was calculated as bone age minus chronological age; negative values indicate skeletal maturation behind chronological age. The dashed horizontal line marks zero deviation.

Boys also had a lower height difference from their corresponding national reference than girls (−2.98 ± 7.69 vs. 0.52 ± 7.71 cm; P = 0.002), whereas measured height did not differ significantly between sexes (118.78 ± 19.93 vs. 121.77 ± 19.60 cm; P = 0.287). Thus, the sex contrast in height difference concerned deviation from the respective sex-specific references. Boys had a higher BMI than girls (17.27 ± 3.26 vs. 16.41 ± 2.85 kg/m²; P = 0.048), but the between-sex difference in BMI difference was not statistically significant (P = 0.202). PTH concentrations were higher in girls than boys (70.98 ± 25.47 vs. 60.32 ± 27.59 pg/mL; P = 0.005; **Table 1**).

### Pubertal-stage distribution

Most participants were classified at Tanner stage I (n = 138, 69.3%), followed by stage II (n = 27, 13.6%), stage III (n = 18, 9.0%), stage IV (n = 14, 7.0%), and stage V (n = 2, 1.0%). Tanner-stage distributions did not differ significantly between girls and boys (P = 0.778; **Table 1**). Tanner stage I included 73 girls and 65 boys, whereas stage V included only one participant of each sex (**Supplementary Figure 1**).

The age distributions within Tanner stages differed descriptively between sexes. At Tanner stage II, median age was 10.17 years in girls and 9.50 years in boys; at stage IV, the corresponding medians were 13.71 and 12.92 years. Among participants at Tanner stage I, mean bone-age deviation was −0.36 ± 1.00 years in girls and −1.09 ± 1.00 years in boys. Detailed age, bone-age-deviation, height-difference, and BMI-difference summaries by sex and Tanner stage are presented in **Supplementary Table 2**.

### Adjusted associations with bone-age deviation and height difference

After adjustment for chronological age and its quadratic term, ethnicity, BMI difference, and pubertal status, boys had a bone-age deviation 0.65 years lower than girls (β = −0.65; 95% CI, −0.96 to −0.33; P < 0.001). Each 1-kg/m² increase in BMI difference was associated with a 0.06-year increase in bone-age deviation (95% CI, 0.01 to 0.10; P = 0.010; **Table 2**). The quadratic age term was positive in the primary bone-age model (P = 0.007), whereas the coefficients for ethnicity and binary pubertal status had confidence intervals spanning zero. Full regression estimates are presented in **Supplementary Table 1**.

**Table 2:** Multivariable associations of sex and BMI difference with bone-age deviation and height difference.

Model	N	Term	Beta (95% CI)	P value
Bone-age deviation	199	Boys vs girls	-0.65 (-0.96 to -0.33)	<0.001
Bone-age deviation	199	BMI difference, per kg/m²	0.06 (0.01 to 0.10)	0.010
Height difference before bone-age adjustment	199	Boys vs girls	-3.39 (-5.63 to -1.15)	0.003
Height difference before bone-age adjustment	199	BMI difference, per kg/m²	-0.34 (-0.77 to 0.10)	0.125
Height difference after bone-age adjustment	199	Bone-age deviation, per year	2.76 (1.82 to 3.70)	<0.001
Height difference after bone-age adjustment	199	Boys vs girls	-1.61 (-3.70 to 0.48)	0.131
Height difference after bone-age adjustment	199	BMI difference, per kg/m²	-0.50 (-0.94 to -0.06)	0.026

All models included 199 participants. Values are unstandardized regression coefficients (β) with 95% confidence intervals (CIs) based on type 3 heteroscedasticity-consistent (HC3) standard errors and the residual degrees of freedom; P values are from two-sided robust t tests. Bone-age deviation was calculated as bone age minus chronological age (years). Height difference and BMI difference were calculated as the measured value minus the corresponding age- and sex-specific national reference mean (cm and kg/m², respectively). Models included sex, mean-centered chronological age and its squared term, ethnicity, BMI difference, and pubertal status (Tanner stage I vs >I); height models after bone-age adjustment additionally included bone-age deviation. Girls, Tibetan ethnicity, and Tanner stage I are the reference categories. Coefficients are expressed in years for the bone-age-deviation outcome and centimeters for the height-difference outcome; continuous exposures are scaled as indicated in the table. The sex coefficient for height difference compares deviations from the respective sex-specific references, not absolute measured height between boys and girls. These cross-sectional coefficients do not establish causal effects. BMI, body mass index.

Unadjusted sex-specific fitted lines showed positive associations between bone-age deviation and height difference (**Figure 2**). In the multivariable model, each 1-year increase in bone-age deviation was associated with a 2.76-cm greater height difference from the national reference (95% CI, 1.82 to 3.70; P < 0.001; **Table 2**).

**Figure 2:**
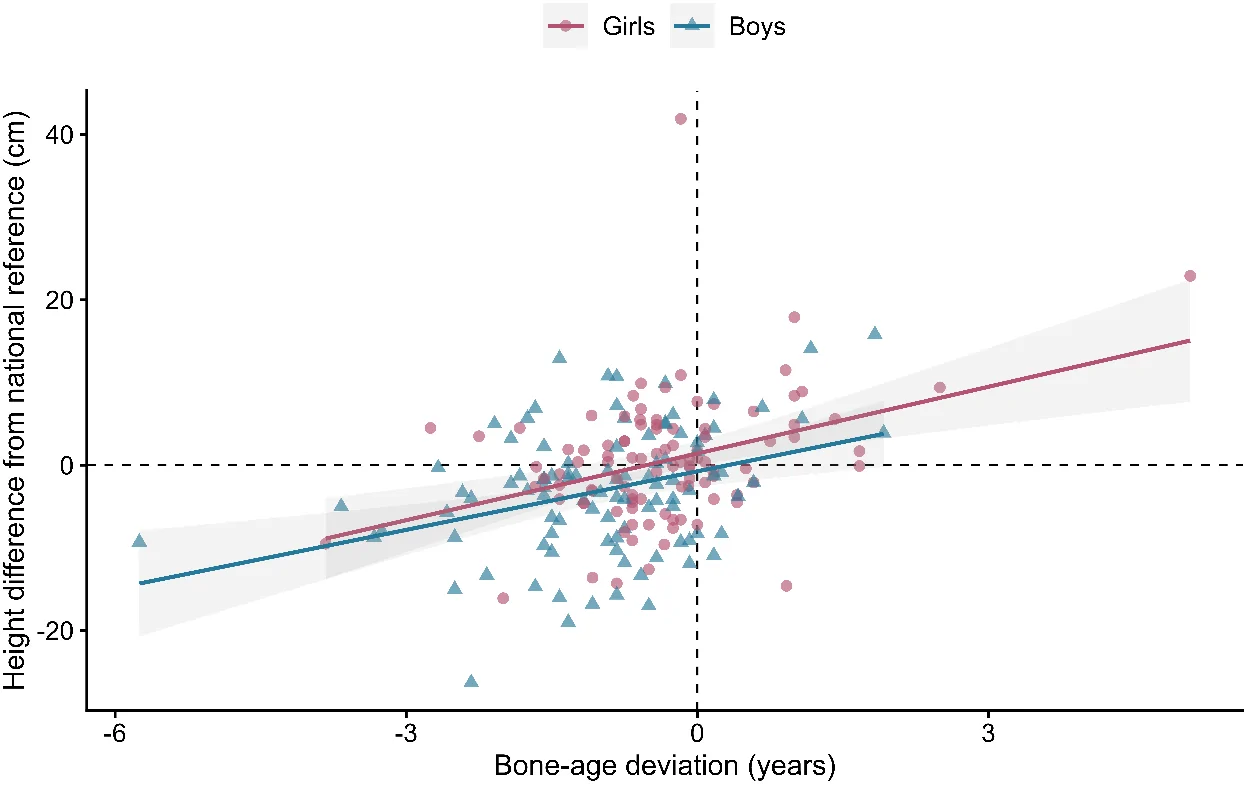
Unadjusted associations between bone-age deviation and height difference from national reference values by sex. Each point represents one of 199 participants: pink circles denote girls (n = 101), and blue triangles denote boys (n = 98). Bone-age deviation was calculated as bone age minus chronological age. Height difference was calculated as measured height minus the age- and sex-specific national reference mean; negative values indicate height below that reference. Solid lines show separate unadjusted ordinary least-squares fits for girls and boys; shaded bands indicate pointwise 95% confidence intervals for the fitted mean. Dashed lines mark zero bone-age deviation and zero height difference. These descriptive fits are distinct from the adjusted associations reported in **Table 2**.

Before adjustment for bone-age deviation, boys had a height difference 3.39 cm lower than girls (95% CI, −5.63 to −1.15; P = 0.003). After bone-age deviation was included, the sex coefficient was attenuated to −1.61 cm, with a confidence interval spanning zero (95% CI, −3.70 to 0.48; P = 0.131). BMI difference was not clearly associated with height difference before bone-age adjustment (β = −0.34 cm per kg/m²; 95% CI, −0.77 to 0.10; P = 0.125), but an inverse association was observed after adjustment (β = −0.50 cm per kg/m²; 95% CI, −0.94 to −0.06; P = 0.026; **Table 2**).

### Sex-related statistical indirect associations

The estimated statistical indirect association between sex and height difference through bone-age deviation was −1.78 cm for boys relative to girls. Both the percentile bootstrap 95% CI (−2.89 to −0.83) and the bias-corrected and accelerated (BCa) 95% CI (−3.02 to −0.90) excluded zero. The direct association was −1.61 cm, with both bootstrap intervals spanning zero, whereas the total association was −3.39 cm, with both intervals excluding zero (**Table 3**).

**Table 3:** Statistical decomposition of the association between sex and height difference through bone-age deviation.

Association	Estimate, cm	Percentile 95% CI	BCa 95% CI
Indirect association	-1.78	-2.89 to -0.83	-3.02 to -0.90
Direct association	-1.61	-3.73 to 0.40	-3.77 to 0.37
Total association	-3.39	-5.57 to -1.22	-5.65 to -1.28

The analysis included 199 participants. Estimates are expressed in centimeters of height difference from the age- and sex-specific national reference mean for boys relative to girls. The indirect association is the product of the sex coefficient in the bone-age-deviation model and the bone-age-deviation coefficient in the height-difference model. The direct association is the sex coefficient after bone-age adjustment; the total association is the sex coefficient before that adjustment. Models were adjusted for mean-centered chronological age and its squared term, ethnicity, BMI difference, and pubertal status (Tanner stage I vs >I). Percentile and bias-corrected and accelerated (BCa) 95% confidence intervals (CIs) were calculated from 5,000 participant-level bootstrap resamples. No proportion mediated is reported. These cross-sectional estimates describe statistical associations and do not establish temporal ordering or causal mediation. BMI, body mass index.

The indirect association was also negative in the Tibetan-only subgroup (−1.92 cm; percentile 95% CI, −3.25 to −0.78), the Tanner stage I subgroup (−1.89 cm; −3.42 to −0.53), and participants aged <10 years (−1.57 cm; −2.93 to −0.45). These estimates and the full-sample estimate are shown in **Figure 3**. The corresponding BCa intervals also excluded zero. Among Tibetan participants, the direct association remained negative, with a percentile 95% CI excluding zero (−2.33 cm; −4.40 to −0.29). The decomposition for participants aged ≥10 years (n = 47) was not estimable under the specified modeling procedure because of unit leverage (**Supplementary Table 4**).

**Figure 3:**
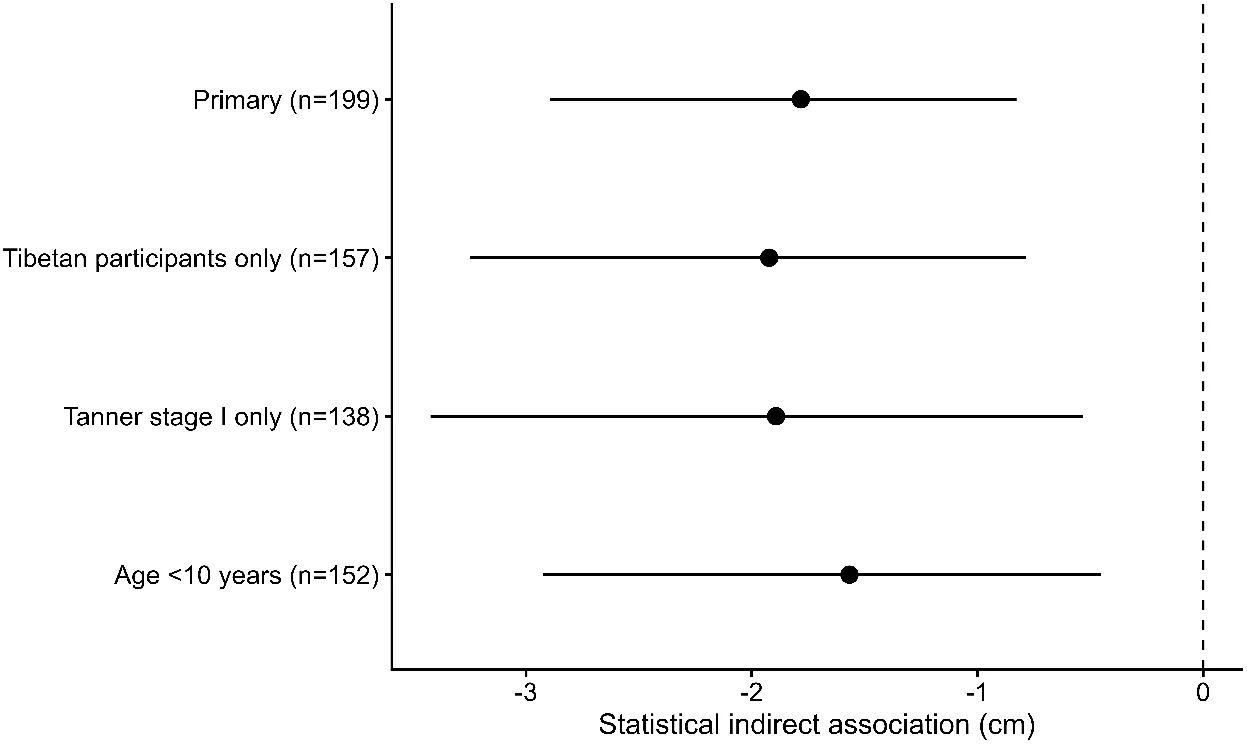
Sex-related statistical indirect associations with height difference through bone-age deviation in the full sample and subgroups. Points show product-of-coefficients estimates of the statistical indirect association for boys relative to girls; horizontal bars show percentile 95% confidence intervals from 5,000 participant-level bootstrap resamples. Estimates are expressed in centimeters of height difference from the age- and sex-specific national reference mean. The analyses include the full sample (n = 199), Tibetan participants (n = 157), participants at Tanner stage I (n = 138), and participants aged <10 years (n = 152). Models were adjusted for mean-centered chronological age and its squared term, ethnicity, BMI difference, and pubertal status (Tanner stage I vs >I), as applicable. Ethnicity was omitted in the Tibetan-only subgroup and pubertal status was omitted in the Tanner stage I subgroup because these variables were constant. The dashed vertical line marks zero. The overlapping subgroups do not constitute formal tests of heterogeneity. Results for participants aged ≥10 years (n = 47) are not plotted because unit leverage prevented reliable estimation under the specified modeling procedure (**Supplementary Table 4**). These cross-sectional estimates describe statistical associations and do not establish temporal ordering or causal mediation. BMI, body mass index.

These estimates describe cross-sectional statistical indirect associations; no proportion mediated was calculated.

### Sensitivity of the principal associations

Replacing binary pubertal status with grouped Tanner stages produced estimates similar to those of the primary analysis. The adjusted sex coefficient for bone-age deviation was −0.66 years (95% CI, −1.00 to −0.32), and the coefficient for bone-age deviation in the height model was 2.77 cm per year (95% CI, 1.83 to 3.71). Omitting BMI adjustment also retained a negative sex association with bone-age deviation (−0.62 years; 95% CI, −0.94 to −0.30) and a positive association between bone-age deviation and height difference (2.55 cm per year; 95% CI, 1.57 to 3.52; **Supplementary Table 3**).

Excluding 21 influential observations left 178 participants. The sex coefficient for bone-age deviation decreased in magnitude to −0.48 years (95% CI, −0.73 to −0.23), and the bone-age-deviation coefficient for height difference decreased to 1.87 cm per year (95% CI, 0.86 to 2.88). Both confidence intervals continued to exclude zero. Exclusion of four participants in the tails of the bone-age-deviation distribution yielded corresponding estimates of −0.56 years (95% CI, −0.82 to −0.30) and 2.69 cm per year (95% CI, 1.51 to 3.86). In contrast, the inverse association between BMI difference and height difference after bone-age adjustment was attenuated following influence exclusion, with its confidence interval spanning zero (β = −0.18 cm per kg/m²; 95% CI, −0.47 to 0.11; **Supplementary Table 3**).

The positive BMI-difference association with bone-age deviation was retained in the grouped-Tanner, influence-exclusion, tail-exclusion, and Tibetan-only analyses. However, confidence intervals spanned zero in participants at Tanner stage I (β = 0.02 years per kg/m²; 95% CI, −0.03 to 0.08) and those aged <10 years (β = 0.03; 95% CI, −0.03 to 0.08; **Supplementary Table 3**).

The sex-related indirect association remained negative with grouped Tanner adjustment (−1.82 cm; percentile 95% CI, −2.97 to −0.86), after influence exclusion (−0.90 cm; −1.63 to −0.32), and after tail exclusion (−1.50 cm; −2.51 to −0.66). BCa intervals also excluded zero in each analysis, although the estimate was smaller after influential observations were removed (**Supplementary Table 4**).

### Exploratory BMI-related statistical indirect associations

For each 1-kg/m² increase in BMI difference, the estimated indirect association with height difference through bone-age deviation was positive (0.16 cm; percentile 95% CI, 0.03 to 0.29; BCa 95% CI, 0.04 to 0.31). The direct association was negative (−0.50 cm; percentile 95% CI, −0.91 to −0.08), whereas the total association had a confidence interval spanning zero (−0.34 cm; −0.76 to 0.08; **Supplementary Table 5**).

Positive indirect estimates were also observed with grouped Tanner adjustment and after exclusion of influential observations or bone-age-deviation tails. After influence exclusion, the indirect estimate was 0.09 cm (percentile 95% CI, 0.02 to 0.17), but the direct estimate was attenuated to −0.18 cm (−0.47 to 0.14). Thus, the indirect and direct components differed in direction, and the direct component was sensitive to influential observations (**Supplementary Table 5**).

### Exploratory biomarker associations

None of the five biomarkers was associated with bone-age deviation at the nominal P < 0.05 level in the mutually adjusted exploratory model. For height difference, 25-hydroxyvitamin D showed an inverse association (β = −0.255 cm per ng/mL; 95% CI, −0.418 to −0.091; P = 0.002), which remained below the FDR threshold after correction across the 10 biomarker–outcome tests (q = 0.025; **Supplementary Table 6**).

ALP was positively associated with height difference before multiplicity correction (β = 0.018 cm per U/L; 95% CI, 0.004 to 0.032; P = 0.010), but its adjusted q value was 0.05047, slightly above the prespecified 0.05 threshold. PTH, calcium, and phosphorus did not show statistically significant associations with height difference (**Supplementary Table 6**).

### Cross-sectional age patterns and interaction tests

The descriptive sex-specific age curves showed a curved relationship between chronological age and bone-age deviation, with fitted values generally more negative in boys than girls and wider confidence intervals toward the older ages represented in the plots (**Supplementary Figure 2**). These curves reflected cross-sectional differences between participants rather than within-person changes over time.

Formal interaction tests did not provide evidence of sex-by-age interaction for bone-age deviation (robust F = 0.666; P = 0.515) or height difference (robust F = 0.327; P = 0.721). The sex-by-BMI-difference interaction for bone-age deviation was also not statistically significant (robust F = 0.109; P = 0.741; **Supplementary Table 7**).

### Regression diagnostics

Heteroscedasticity tests were significant for the height-difference models before and after bone-age adjustment (P = 0.001 and P = 0.002, respectively), but not for the primary bone-age-deviation model (P = 0.759). Maximum Cook’s distances exceeded the sensitivity-analysis threshold in both the primary bone-age model and the bone-age-adjusted height model (**Supplementary Table 8**).

Residual-versus-fitted plots showed observations with large standardized residuals (**Supplementary Figure 3**), and normal quantile–quantile plots showed departures from the reference lines in the distribution tails (**Supplementary Figure 4**). Regression estimates were reported using HC3 standard errors, and the influence-exclusion analyses described above assessed the sensitivity of the principal findings.

## Discussion

This study identified three related findings in children and adolescents living in Lhasa. Boys had greater bone-age delay and a more negative height difference from their corresponding national reference than girls. Greater bone-age deviation was associated with greater height relative to the age- and sex-specific reference, and higher BMI difference was associated with more advanced skeletal maturation. The sex-related associations with bone-age deviation and the association between bone-age deviation and height remained evident across several sensitivity analyses. However, the BMI-related findings were less consistent across subgroups and after exclusion of influential observations. Together, these results support considering skeletal maturation alongside current height in this clinical sample, while leaving the causes and long-term consequences of the observed differences unresolved.

The sex-related finding requires careful interpretation because height difference was calculated against separate reference values for boys and girls. The adjusted difference of −3.39 cm therefore means that boys were further below their corresponding male reference than girls were below their female reference; it is not a direct comparison of absolute height between the sexes. After adjustment for bone-age deviation, this estimate decreased to −1.61 cm, with a confidence interval that included zero. This attenuation suggests that skeletal maturation contributes useful information when interpreting the observed sex difference. Nevertheless, an association compatible with zero does not demonstrate the absence of a remaining sex difference. In the Tibetan-only analysis, the adjusted sex coefficient remained negative and its confidence interval excluded zero. Bone-age deviation therefore did not fully account for the sex-related pattern in every analysis.

Pubertal development is an important consideration in interpreting these findings. Longitudinal observations have demonstrated substantial variation in the timing and progression of puberty, including variation in the relationship between pubertal stage and the adolescent growth spurt (11). In the present study, replacing binary pubertal status with grouped Tanner stages produced similar principal estimates. The sex difference in bone-age deviation also remained evident among participants at Tanner stage I, suggesting that it was not confined to children with clinically apparent puberty. However, neither analysis eliminates developmental confounding. Tanner stage does not measure time since pubertal onset, and children within the same stage may differ in their hormonal development. Furthermore, most participants were prepubertal, later Tanner stages were sparsely represented, and the fully adjusted decomposition could not be reliably estimated in participants aged ≥10 years. The findings therefore describe the younger and prepubertal participants more precisely than adolescents in later puberty.

The positive association between bone-age deviation and height difference indicates that children with relatively more advanced skeletal maturation tended to be taller relative to their age- and sex-specific reference. The estimated difference of 2.76 cm per year of bone-age deviation is a between-child association, not an estimate of height gained by an individual as bone age advances. Similarly, the negative statistical indirect association linking male sex, bone-age deviation, and height describes how these measurements covaried within the sample. Cross-sectional mediation estimates can differ substantially from the corresponding longitudinal relationships, even under apparently favorable modeling conditions (12). Our findings consequently do not establish that skeletal delay caused the observed height differences, nor do they show whether children with delayed bone age will subsequently experience catch-up growth or attain a different adult height.

The association between higher BMI difference and more advanced skeletal maturation is consistent with evidence from other pediatric populations. A Chinese study found an association between overweight or obesity and accelerated skeletal maturation as early as preschool age (13). In children with obesity, de Groot and colleagues also identified an independent association between dehydroepiandrosterone sulfate and advanced bone age, suggesting that adrenal androgen activity may be relevant to the relationship between body size and maturation (14). These studies provide biological context, but the relevant hormonal pathways were not measured adequately in our analysis. Moreover, the BMI–bone-age association was weaker and imprecisely estimated in the Tanner stage I and younger-than-10-year subgroups. These subgroup results do not establish an age- or puberty-specific difference, but they limit claims that the association was uniform throughout childhood.

The BMI-related decomposition warrants additional caution. Its positive indirect association and negative direct association should not be interpreted as evidence that higher BMI simultaneously promotes and inhibits growth through separate biological mechanisms. BMI includes height in its denominator: for a given weight, a shorter child necessarily has a higher BMI. Subtracting an age- and sex-specific BMI reference does not remove this mathematical connection with height. In addition, the negative direct association weakened after influential observations were excluded, and its confidence interval then included zero. The total BMI association was also imprecisely estimated. These features support retaining the BMI decomposition as exploratory. Repeated measurements of growth and body composition would be more informative for separating adiposity, lean mass, and maturational timing.

The high-altitude setting is relevant to the interpretation of the findings, but altitude itself was not evaluated as a causal exposure. Previous research in Tibetan children younger than 36 months reported an association between residential altitude and impaired linear growth after accounting for measured family, feeding, and health-related factors (15). That study provides contextual evidence, although its age range and outcome differ from ours. Population-specific adaptation also complicates a simple interpretation based on oxygen availability. For example, variants near EPAS1 have been associated with lower hemoglobin concentrations in Tibetan highlanders (16). This evidence concerns adaptation of oxygen-related physiology and does not demonstrate an effect on skeletal maturation in our participants. Without a contemporaneous low-altitude comparison group, exposure histories, or physiological measures of hypoxia, the present study cannot distinguish the contribution of altitude from nutrition, socioeconomic circumstances, ancestry, or other shared influences.

Genetic variation offers a further context for skeletal development. Studies of osteogenesis imperfecta in Vietnamese, Estonian, and Ukrainian patients have documented pathogenic variants in COL1A1 and COL1A2 and variation in their clinical manifestations (8–10). More recently, PLOD2-related Bruck syndrome was described in Indonesian and Ukrainian patients, illustrating how abnormalities in collagen processing can compromise skeletal integrity (17). These findings establish the relevance of collagen structure and processing to inherited skeletal disease. They do not, however, show that collagen variants explain the modest differences in BMI, bone age, or height observed in this sample. No genetic measurements were available, and skeletal maturity should not be equated with bone strength or bone mass. Assigning the present associations to specific collagen genes would therefore exceed the available evidence.

Population structure also deserves attention when interpreting differences from national references. Research on the genetics of height has shown that residual population stratification can exaggerate apparent signals of polygenic adaptation (18). Although our study did not analyze genetic associations, this finding reinforces the need to distinguish observed population differences from genetic explanations. Adjustment for Tibetan versus Han ethnicity, and restriction to Tibetan participants, cannot account fully for genetic ancestry or for environmental variation within either group. National reference values remain useful descriptive benchmarks, but their applicability to this clinical sample may be affected by differences in body proportions, living conditions, and population composition. A negative deviation from a reference mean alone should therefore not be interpreted as evidence of a specific environmental injury or inherited abnormality.

The exploratory biochemical analyses did not identify associations with bone-age deviation that survived correction for multiple testing. Higher 25-hydroxyvitamin D was associated with lower height difference in the adjusted height model, whereas the positive alkaline phosphatase association was borderline after correction. The vitamin D finding should not be interpreted as evidence that vitamin D impairs growth. A single circulating measurement may reflect recent exposure or supplementation and cannot reconstruct the nutritional history underlying attained height. In a large randomized trial among Mongolian schoolchildren, vitamin D supplementation increased circulating 25-hydroxyvitamin D concentrations without altering linear growth over three years (19). That trial does not explain the inverse association in our sample, but it underscores the distinction between a biomarker association and an intervention effect. These biochemical findings require independent confirmation.

Strengths of this study include the joint evaluation of anthropometry and skeletal maturation, analysis of bone-age deviation as a continuous measure, allowance for nonlinear age associations, and the use of heteroscedasticity-consistent inference and bootstrap confidence intervals. Detailed pubertal descriptions and several sensitivity analyses helped assess the dependence of the findings on modeling choices and influential observations. Nevertheless, the single-center clinical sample was not designed to represent all children living in Xizang. The cross-sectional design precluded assessment of growth velocity, temporal ordering, or adult height. Important potential confounders were unavailable, including parental height, detailed nutritional and socioeconomic information, duration of altitude exposure, oxygen saturation, hemoglobin concentration, IGF-1, insulin, leptin, and detailed sex-steroid measurements. Residual and unmeasured confounding therefore remains possible. In addition, formal inter-reader and intra-reader reliability estimates for bone-age assessment were unavailable for the present analysis, leaving some uncertainty regarding measurement reproducibility. Statistical adjustment and sensitivity analyses cannot eliminate these limitations.

The practical implication is that current stature and skeletal maturation provide complementary information in this setting. A child who is below the national height reference may also be less skeletally mature, but the present data cannot determine that child’s subsequent growth trajectory or eventual adult height. Prospective studies incorporating serial measurements of height and bone age, detailed pubertal assessment, parental height, body composition, endocrine markers, and environmental information are needed to clarify these relationships. Comparisons across altitude settings should use consistent assessment methods and account for ancestry, living conditions, and other population-specific factors. Within the limits of the present study, the most clearly supported findings are greater skeletal maturation delay among boys, an association between skeletal maturation and height relative to national reference values, and an exploratory positive association between BMI difference and skeletal maturation.

## Conclusion

Among children and adolescents living in Lhasa, boys showed greater skeletal maturation delay than girls, and bone-age deviation was strongly associated with current height relative to age- and sex-specific national reference values. Higher BMI relative to the corresponding reference was associated with more advanced skeletal maturation, although the BMI-related direct and indirect associations with height were less consistent and should be considered exploratory. The sex-related statistical indirect association indicates that skeletal maturation contributes information to the concurrent relationship between sex and height difference, but the cross-sectional design does not establish temporal or causal mediation. Furthermore, because the study did not include a contemporaneous low-altitude comparison group, the observed differences cannot be attributed specifically to altitude or chronic hypoxia. Longitudinal studies incorporating repeated measurements of height and skeletal maturity, detailed pubertal assessment, parental height, body composition, environmental exposures, and population background are needed to clarify the developmental and clinical significance of these findings.

## Data Availability

The raw data supporting the conclusions of this article will be made available by the authors, without undue reservation.
