# Supplementary material for: Sex, BMI, skeletal maturation, and height among children and adolescents in high-altitude Xizang: a cross-sectional study
Source: Front Endocrinol (Lausanne). 2026 Oct 6;17:1942630. doi: 10.3389/fendo.2026.1942630 (PMC13680247; doi:10.3389/fendo.2026.1942630)
Supplement: Supplementary file 1 [file SupplementaryFile1.docx]

**Supplementary Figures**

**Supplementary Figure 1. Distribution of participants across detailed Tanner stages by sex.**

Number of girls and boys classified at Tanner stages I–V. Bars show the number of participants in each sex-specific Tanner-stage category. Tanner stage V was sparse and was therefore not fitted as a separate category in multivariable regression models. Detailed Tanner-stage distributions are presented to characterize pubertal heterogeneity in the study population. The sample comprised 101 girls and 98 boys. Pink bars denote girls and blue bars denote boys; numbers above the bars give the corresponding counts. Tanner stage I denotes prepubertal status. Tanner stage V included one participant of each sex.

**Supplementary Figure 2. Sex-specific cross-sectional associations between chronological age and bone-age deviation.**

Curves show model-based mean bone-age deviation for girls (pink) and boys (blue) in the analytical sample (n = 199). The model included sex, mean-centered chronological age and its squared term, sex interactions with both age terms, ethnicity, and BMI difference. Predictions were averaged over the observed ethnicity and BMI-difference distribution of the full analytical sample and plotted from the 2.5th to the 97.5th percentile of age within each sex. Shaded bands indicate pointwise 95% confidence intervals calculated using the HC3 covariance matrix and a t critical value based on the residual degrees of freedom. Rug marks show observed ages by sex. Tanner stage was omitted from this descriptive age model. Bone-age deviation was calculated as bone age minus chronological age; negative values indicate skeletal maturation behind chronological age. These curves describe between-participant age associations, not longitudinal growth trajectories. BMI, body mass index; HC3, type 3 heteroscedasticity-consistent covariance estimator.

**Supplementary Figure 3. Residual-versus-fitted plots for the principal height-difference and bone-age-deviation models.**

Standardized residuals are plotted against fitted values for the height-difference model after bone-age adjustment (left) and the primary bone-age-deviation model (right), each fitted in 199 participants. Each point represents one participant, and dashed horizontal lines mark zero residuals. Fitted values are expressed in centimeters for height difference and years for bone-age deviation; standardized residuals are dimensionless. Models included sex, mean-centered chronological age and its squared term, ethnicity, BMI difference, and pubertal status (Tanner stage I vs >I); height models after bone-age adjustment additionally included bone-age deviation. The plots assess residual patterns and nonconstant variance; numerical diagnostics are reported in Supplementary Table 8. BMI, body mass index.

**Supplementary Figure 4. Normal quantile–quantile plots of residuals from the principal height-difference and bone-age-deviation models.**

The plots compare standardized residuals with theoretical standard-normal quantiles for the height-difference model after bone-age adjustment (left) and the primary bone-age-deviation model (right), each fitted in 199 participants. Points represent ordered standardized residuals. Reference lines pass through the first and third quartiles of the theoretical and observed distributions; departures from these lines indicate deviations from normality. Models included sex, mean-centered chronological age and its squared term, ethnicity, BMI difference, and pubertal status (Tanner stage I vs >I); height models after bone-age adjustment additionally included bone-age deviation. These graphical diagnostics complement Supplementary Figure 3 and Supplementary Table 8. BMI, body mass index.

**Supplementary Tables**

**Supplementary Table 1. Full HC3 regression estimates for the primary, sensitivity, and interaction models.**

| **Model** | **N** | **Term** | **Beta (95% CI)** | **P value** |
| --- | --- | --- | --- | --- |
| Primary bone-age model | 199 | Intercept | -0.72 (-1.03 to -0.40) | <0.001 |
| Primary bone-age model | 199 | Boys vs girls | -0.65 (-0.96 to -0.33) | <0.001 |
| Primary bone-age model | 199 | Age, centered, years | -0.06 (-0.16 to 0.03) | 0.159 |
| Primary bone-age model | 199 | Centered age squared | 0.02 (0.01 to 0.04) | 0.007 |
| Primary bone-age model | 199 | Han vs Tibetan ethnicity | 0.15 (-0.27 to 0.56) | 0.483 |
| Primary bone-age model | 199 | BMI difference, per kg/m² | 0.06 (0.01 to 0.10) | 0.010 |
| Primary bone-age model | 199 | Tanner >I vs Tanner I | 0.26 (-0.36 to 0.89) | 0.409 |
| Height model before bone-age adjustment | 199 | Intercept | -0.56 (-2.62 to 1.51) | 0.596 |
| Height model before bone-age adjustment | 199 | Boys vs girls | -3.39 (-5.63 to -1.15) | 0.003 |
| Height model before bone-age adjustment | 199 | Age, centered, years | -0.48 (-1.32 to 0.36) | 0.263 |
| Height model before bone-age adjustment | 199 | Centered age squared | 0.01 (-0.14 to 0.16) | 0.894 |
| Height model before bone-age adjustment | 199 | Han vs Tibetan ethnicity | 2.60 (-0.93 to 6.13) | 0.148 |
| Height model before bone-age adjustment | 199 | BMI difference, per kg/m² | -0.34 (-0.77 to 0.10) | 0.125 |
| Height model before bone-age adjustment | 199 | Tanner >I vs Tanner I | 2.35 (-3.87 to 8.57) | 0.457 |
| Height model after bone-age adjustment | 199 | Intercept | 1.42 (-0.56 to 3.41) | 0.159 |
| Height model after bone-age adjustment | 199 | Bone-age deviation, per year | 2.76 (1.82 to 3.70) | <0.001 |
| Height model after bone-age adjustment | 199 | Boys vs girls | -1.61 (-3.70 to 0.48) | 0.131 |
| Height model after bone-age adjustment | 199 | Age, centered, years | -0.30 (-1.08 to 0.48) | 0.450 |
| Height model after bone-age adjustment | 199 | Centered age squared | -0.06 (-0.20 to 0.08) | 0.426 |
| Height model after bone-age adjustment | 199 | Han vs Tibetan ethnicity | 2.19 (-1.08 to 5.47) | 0.188 |
| Height model after bone-age adjustment | 199 | BMI difference, per kg/m² | -0.50 (-0.94 to -0.06) | 0.026 |
| Height model after bone-age adjustment | 199 | Tanner >I vs Tanner I | 1.63 (-4.17 to 7.42) | 0.580 |
| Bone-age model: grouped Tanner sensitivity | 199 | Intercept | -0.70 (-1.03 to -0.37) | <0.001 |
| Bone-age model: grouped Tanner sensitivity | 199 | Boys vs girls | -0.66 (-1.00 to -0.32) | <0.001 |
| Bone-age model: grouped Tanner sensitivity | 199 | Age, centered, years | -0.07 (-0.17 to 0.03) | 0.165 |
| Bone-age model: grouped Tanner sensitivity | 199 | Centered age squared | 0.02 (-0.01 to 0.05) | 0.123 |
| Bone-age model: grouped Tanner sensitivity | 199 | Han vs Tibetan ethnicity | 0.13 (-0.28 to 0.55) | 0.523 |
| Bone-age model: grouped Tanner sensitivity | 199 | BMI difference, per kg/m² | 0.06 (0.01 to 0.10) | 0.009 |
| Bone-age model: grouped Tanner sensitivity | 199 | Tanner II-III vs Tanner I | 0.29 (-0.36 to 0.94) | 0.383 |
| Bone-age model: grouped Tanner sensitivity | 199 | Tanner IV-V vs Tanner I | 0.52 (-0.85 to 1.88) | 0.457 |
| Height model: grouped Tanner sensitivity | 199 | Intercept | 1.33 (-0.70 to 3.37) | 0.198 |
| Height model: grouped Tanner sensitivity | 199 | Bone-age deviation, per year | 2.77 (1.83 to 3.71) | <0.001 |
| Height model: grouped Tanner sensitivity | 199 | Boys vs girls | -1.53 (-3.74 to 0.67) | 0.171 |
| Height model: grouped Tanner sensitivity | 199 | Age, centered, years | -0.27 (-1.09 to 0.55) | 0.515 |
| Height model: grouped Tanner sensitivity | 199 | Centered age squared | -0.04 (-0.22 to 0.15) | 0.710 |
| Height model: grouped Tanner sensitivity | 199 | Han vs Tibetan ethnicity | 2.26 (-0.99 to 5.52) | 0.172 |
| Height model: grouped Tanner sensitivity | 199 | BMI difference, per kg/m² | -0.51 (-0.95 to -0.06) | 0.025 |
| Height model: grouped Tanner sensitivity | 199 | Tanner II-III vs Tanner I | 1.48 (-4.53 to 7.49) | 0.628 |
| Height model: grouped Tanner sensitivity | 199 | Tanner IV-V vs Tanner I | 0.20 (-10.20 to 10.59) | 0.970 |
| Bone-age model: no BMI adjustment | 199 | Intercept | -0.67 (-0.99 to -0.36) | <0.001 |
| Bone-age model: no BMI adjustment | 199 | Boys vs girls | -0.62 (-0.94 to -0.30) | <0.001 |
| Bone-age model: no BMI adjustment | 199 | Age, centered, years | -0.07 (-0.16 to 0.02) | 0.126 |
| Bone-age model: no BMI adjustment | 199 | Centered age squared | 0.03 (0.01 to 0.04) | 0.006 |
| Bone-age model: no BMI adjustment | 199 | Han vs Tibetan ethnicity | 0.10 (-0.32 to 0.51) | 0.651 |
| Bone-age model: no BMI adjustment | 199 | Tanner >I vs Tanner I | 0.26 (-0.38 to 0.90) | 0.424 |
| Height model: no BMI adjustment | 199 | Intercept | 0.88 (-1.24 to 2.99) | 0.416 |
| Height model: no BMI adjustment | 199 | Bone-age deviation, per year | 2.55 (1.57 to 3.52) | <0.001 |
| Height model: no BMI adjustment | 199 | Boys vs girls | -1.99 (-4.17 to 0.20) | 0.074 |
| Height model: no BMI adjustment | 199 | Age, centered, years | -0.26 (-1.07 to 0.54) | 0.518 |
| Height model: no BMI adjustment | 199 | Centered age squared | -0.06 (-0.20 to 0.08) | 0.410 |
| Height model: no BMI adjustment | 199 | Han vs Tibetan ethnicity | 2.67 (-0.81 to 6.14) | 0.132 |
| Height model: no BMI adjustment | 199 | Tanner >I vs Tanner I | 1.69 (-4.35 to 7.73) | 0.581 |
| Bone-age model: sex by BMI interaction | 199 | Intercept | -0.71 (-1.04 to -0.37) | <0.001 |
| Bone-age model: sex by BMI interaction | 199 | Boys vs girls | -0.66 (-1.00 to -0.32) | <0.001 |
| Bone-age model: sex by BMI interaction | 199 | Age, centered, years | -0.06 (-0.15 to 0.03) | 0.167 |
| Bone-age model: sex by BMI interaction | 199 | Centered age squared | 0.02 (0.01 to 0.04) | 0.007 |
| Bone-age model: sex by BMI interaction | 199 | Han vs Tibetan ethnicity | 0.14 (-0.27 to 0.56) | 0.498 |
| Bone-age model: sex by BMI interaction | 199 | BMI difference, per kg/m² | 0.05 (-0.03 to 0.13) | 0.223 |
| Bone-age model: sex by BMI interaction | 199 | Tanner >I vs Tanner I | 0.25 (-0.37 to 0.88) | 0.426 |
| Bone-age model: sex by BMI interaction | 199 | Sex × BMI difference | 0.01 (-0.07 to 0.10) | 0.741 |
| Bone-age model: sex by age interaction | 199 | Intercept | -0.60 (-0.99 to -0.22) | 0.002 |
| Bone-age model: sex by age interaction | 199 | Boys vs girls | -0.90 (-1.49 to -0.31) | 0.003 |
| Bone-age model: sex by age interaction | 199 | Age, centered, years | -0.06 (-0.16 to 0.04) | 0.270 |
| Bone-age model: sex by age interaction | 199 | Centered age squared | 0.01 (-0.03 to 0.05) | 0.564 |
| Bone-age model: sex by age interaction | 199 | Han vs Tibetan ethnicity | 0.10 (-0.35 to 0.54) | 0.669 |
| Bone-age model: sex by age interaction | 199 | BMI difference, per kg/m² | 0.06 (0.01 to 0.10) | 0.015 |
| Bone-age model: sex by age interaction | 199 | Tanner II-III vs Tanner I | 0.34 (-0.38 to 1.07) | 0.349 |
| Bone-age model: sex by age interaction | 199 | Tanner IV-V vs Tanner I | 0.54 (-0.97 to 2.04) | 0.482 |
| Bone-age model: sex by age interaction | 199 | Sex × centered age | -0.05 (-0.20 to 0.10) | 0.521 |
| Bone-age model: sex by age interaction | 199 | Sex × centered age squared | 0.03 (-0.02 to 0.07) | 0.254 |
| Height model: sex by age interaction | 199 | Intercept | 1.31 (-0.88 to 3.50) | 0.239 |
| Height model: sex by age interaction | 199 | Bone-age deviation, per year | 2.77 (1.82 to 3.71) | <0.001 |
| Height model: sex by age interaction | 199 | Boys vs girls | -1.34 (-5.31 to 2.62) | 0.504 |
| Height model: sex by age interaction | 199 | Age, centered, years | -0.39 (-1.16 to 0.39) | 0.327 |
| Height model: sex by age interaction | 199 | Centered age squared | -0.02 (-0.24 to 0.19) | 0.836 |
| Height model: sex by age interaction | 199 | Han vs Tibetan ethnicity | 2.24 (-1.01 to 5.48) | 0.175 |
| Height model: sex by age interaction | 199 | BMI difference, per kg/m² | -0.49 (-0.96 to -0.01) | 0.043 |
| Height model: sex by age interaction | 199 | Tanner II-III vs Tanner I | 1.20 (-5.22 to 7.61) | 0.713 |
| Height model: sex by age interaction | 199 | Tanner IV-V vs Tanner I | -0.46 (-11.67 to 10.75) | 0.936 |
| Height model: sex by age interaction | 199 | Sex × centered age | 0.35 (-0.68 to 1.38) | 0.509 |
| Height model: sex by age interaction | 199 | Sex × centered age squared | -0.02 (-0.33 to 0.29) | 0.911 |

**Note.** Values are unstandardized regression coefficients (β) with HC3 heteroscedasticity-consistent 95% confidence intervals and two-sided P values. Primary models included sex, centered chronological age, centered age squared, ethnicity, BMI difference, and Tanner stage I vs >I, as applicable; height models estimating direct associations additionally included bone-age deviation. Grouped-Tanner sensitivity models used Tanner I, Tanner II–III, and Tanner IV–V. Interaction models additionally included the specified sex-by-age or sex-by-BMI terms. Girls, Tibetan ethnicity, and Tanner stage I were the reference categories. BMI, body mass index; CI, confidence interval; HC3, heteroscedasticity-consistent covariance estimator type 3. Bone-age deviation was calculated as bone age minus chronological age (years). Height difference and BMI difference were calculated as the measured value minus the corresponding age- and sex-specific national reference mean (cm and kg/m², respectively). Coefficients are in years for bone-age deviation and centimeters for height difference. The squared age term is the square of mean-centered age. Sex-by-age models used grouped Tanner stages (I, II–III, and IV–V); the sex-by-BMI model used Tanner I vs >I. Models labeled “no BMI adjustment” omitted BMI difference. N denotes the number of participants included in each model.

**Supplementary Table 2. Participant characteristics by sex and detailed Tanner stage.**

| **Sex** | **Tanner stage** | **N** | **Age, median [IQR], years** | **Age range, years** | **Bone-age deviation, mean (SD), years** | **Height difference, mean (SD), cm** | **BMI difference, mean (SD), kg/m²** |
| --- | --- | --- | --- | --- | --- | --- | --- |
| Girls | I | 73 | 5.58 [4.42, 6.67] | 3.50 to 11.00 | -0.36 (1.00) | 0.08 (6.42) | 0.86 (2.63) |
| Girls | II | 11 | 10.17 [7.83, 10.66] | 6.58 to 11.00 | -0.58 (1.60) | 5.88 (14.10) | -0.35 (2.74) |
| Girls | III | 10 | 11.96 [11.92, 12.46] | 10.00 to 12.92 | -0.01 (1.00) | -0.25 (4.16) | 2.28 (3.23) |
| Girls | IV | 6 | 13.71 [13.52, 14.02] | 13.42 to 14.25 | -0.43 (0.47) | -2.90 (9.22) | -1.00 (1.16) |
| Girls | V | 1 | 15.33 [15.33, 15.33] | 15.33 to 15.33 | 1.67 (NA) | 1.70 (NA) | 0.33 (NA) |
| Boys | I | 65 | 5.33 [4.83, 6.08] | 3.25 to 8.92 | -1.09 (1.00) | -3.07 (6.76) | 1.56 (3.31) |
| Boys | II | 16 | 9.50 [7.25, 10.50] | 6.50 to 11.75 | -1.21 (1.40) | -6.95 (7.58) | 0.67 (2.74) |
| Boys | III | 8 | 11.62 [11.06, 12.08] | 10.00 to 13.00 | -0.25 (1.35) | 2.35 (10.51) | 1.72 (3.97) |
| Boys | IV | 8 | 12.92 [12.81, 13.33] | 12.58 to 15.08 | 0.03 (0.65) | 0.40 (9.25) | 0.66 (2.60) |
| Boys | V | 1 | 14.33 [14.33, 14.33] | 14.33 to 14.33 | -0.83 (NA) | -3.90 (NA) | -3.87 (NA) |

**Note.** All five Tanner stages are presented descriptively for girls and boys. Age is presented as median [25th percentile, 75th percentile] and range; bone-age deviation, height difference, and BMI difference are presented as mean (SD). SD is not estimable for strata containing a single participant. Tanner stage V was sparse, with one girl and one boy, and was therefore not modeled as an independent multivariable stratum. These descriptive data characterize pubertal heterogeneity rather than provide formal comparisons across individual Tanner stages. BMI, body mass index; IQR, interquartile range; SD, standard deviation; NA, not available because the stratum contains only one participant. Bone-age deviation was calculated as bone age minus chronological age (years). Height difference and BMI difference were calculated as the measured value minus the corresponding age- and sex-specific national reference mean (cm and kg/m², respectively). N denotes the number of participants in each sex-by-stage stratum.

**Supplementary Table 3. Sensitivity and subgroup analyses of the principal regression associations.**

| **Model** | **N** | **Term** | **Beta (95% CI)** | **P value** |
| --- | --- | --- | --- | --- |
| Primary \| bone-age deviation | 199 | Boys vs girls | -0.65 (-0.96 to -0.33) | <0.001 |
| Primary \| bone-age deviation | 199 | BMI difference, per kg/m² | 0.06 (0.01 to 0.10) | 0.010 |
| Primary \| height after bone-age adjustment | 199 | Bone-age deviation, per year | 2.76 (1.82 to 3.70) | <0.001 |
| Primary \| height after bone-age adjustment | 199 | Boys vs girls | -1.61 (-3.70 to 0.48) | 0.131 |
| Primary \| height after bone-age adjustment | 199 | BMI difference, per kg/m² | -0.50 (-0.94 to -0.06) | 0.026 |
| Primary \| height before bone-age adjustment | 199 | Boys vs girls | -3.39 (-5.63 to -1.15) | 0.003 |
| Primary \| height before bone-age adjustment | 199 | BMI difference, per kg/m² | -0.34 (-0.77 to 0.10) | 0.125 |
| Grouped Tanner sensitivity \| bone-age deviation | 199 | Boys vs girls | -0.66 (-1.00 to -0.32) | <0.001 |
| Grouped Tanner sensitivity \| bone-age deviation | 199 | BMI difference, per kg/m² | 0.06 (0.01 to 0.10) | 0.009 |
| Grouped Tanner sensitivity \| height after bone-age adjustment | 199 | Bone-age deviation, per year | 2.77 (1.83 to 3.71) | <0.001 |
| Grouped Tanner sensitivity \| height after bone-age adjustment | 199 | Boys vs girls | -1.53 (-3.74 to 0.67) | 0.171 |
| Grouped Tanner sensitivity \| height after bone-age adjustment | 199 | BMI difference, per kg/m² | -0.51 (-0.95 to -0.06) | 0.025 |
| Grouped Tanner sensitivity \| height before bone-age adjustment | 199 | Boys vs girls | -3.35 (-5.70 to -1.01) | 0.005 |
| Grouped Tanner sensitivity \| height before bone-age adjustment | 199 | BMI difference, per kg/m² | -0.34 (-0.78 to 0.09) | 0.123 |
| No BMI adjustment \| bone-age deviation | 199 | Boys vs girls | -0.62 (-0.94 to -0.30) | <0.001 |
| No BMI adjustment \| height after bone-age adjustment | 199 | Bone-age deviation, per year | 2.55 (1.57 to 3.52) | <0.001 |
| No BMI adjustment \| height after bone-age adjustment | 199 | Boys vs girls | -1.99 (-4.17 to 0.20) | 0.074 |
| No BMI adjustment \| height before bone-age adjustment | 199 | Boys vs girls | -3.56 (-5.88 to -1.24) | 0.003 |
| Cook-influential observations excluded \| bone-age deviation | 178 | Boys vs girls | -0.48 (-0.73 to -0.23) | <0.001 |
| Cook-influential observations excluded \| bone-age deviation | 178 | BMI difference, per kg/m² | 0.05 (0.01 to 0.08) | 0.009 |
| Cook-influential observations excluded \| height after bone-age adjustment | 178 | Bone-age deviation, per year | 1.87 (0.86 to 2.88) | <0.001 |
| Cook-influential observations excluded \| height after bone-age adjustment | 178 | Boys vs girls | -1.31 (-3.12 to 0.50) | 0.154 |
| Cook-influential observations excluded \| height after bone-age adjustment | 178 | BMI difference, per kg/m² | -0.18 (-0.47 to 0.11) | 0.219 |
| Cook-influential observations excluded \| height before bone-age adjustment | 178 | Boys vs girls | -2.21 (-4.00 to -0.42) | 0.016 |
| Cook-influential observations excluded \| height before bone-age adjustment | 178 | BMI difference, per kg/m² | -0.09 (-0.38 to 0.19) | 0.509 |
| Bone-age 1st/99th percentile tails excluded \| bone-age deviation | 195 | Boys vs girls | -0.56 (-0.82 to -0.30) | <0.001 |
| Bone-age 1st/99th percentile tails excluded \| bone-age deviation | 195 | BMI difference, per kg/m² | 0.06 (0.02 to 0.09) | 0.001 |
| Bone-age 1st/99th percentile tails excluded \| height after bone-age adjustment | 195 | Bone-age deviation, per year | 2.69 (1.51 to 3.86) | <0.001 |
| Bone-age 1st/99th percentile tails excluded \| height after bone-age adjustment | 195 | Boys vs girls | -1.65 (-3.81 to 0.52) | 0.135 |
| Bone-age 1st/99th percentile tails excluded \| height after bone-age adjustment | 195 | BMI difference, per kg/m² | -0.48 (-0.93 to -0.03) | 0.035 |
| Bone-age 1st/99th percentile tails excluded \| height before bone-age adjustment | 195 | Boys vs girls | -3.15 (-5.38 to -0.92) | 0.006 |
| Bone-age 1st/99th percentile tails excluded \| height before bone-age adjustment | 195 | BMI difference, per kg/m² | -0.32 (-0.75 to 0.11) | 0.138 |
| Tibetan participants only \| bone-age deviation | 157 | Boys vs girls | -0.83 (-1.19 to -0.46) | <0.001 |
| Tibetan participants only \| bone-age deviation | 157 | BMI difference, per kg/m² | 0.05 (0.01 to 0.10) | 0.024 |
| Tibetan participants only \| height after bone-age adjustment | 157 | Bone-age deviation, per year | 2.32 (1.23 to 3.41) | <0.001 |
| Tibetan participants only \| height after bone-age adjustment | 157 | Boys vs girls | -2.33 (-4.44 to -0.22) | 0.030 |
| Tibetan participants only \| height after bone-age adjustment | 157 | BMI difference, per kg/m² | -0.40 (-0.79 to -0.02) | 0.039 |
| Tibetan participants only \| height before bone-age adjustment | 157 | Boys vs girls | -4.25 (-6.41 to -2.10) | <0.001 |
| Tibetan participants only \| height before bone-age adjustment | 157 | BMI difference, per kg/m² | -0.28 (-0.65 to 0.08) | 0.127 |
| Tanner stage I only \| bone-age deviation | 138 | Boys vs girls | -0.80 (-1.14 to -0.45) | <0.001 |
| Tanner stage I only \| bone-age deviation | 138 | BMI difference, per kg/m² | 0.02 (-0.03 to 0.08) | 0.400 |
| Tanner stage I only \| height after bone-age adjustment | 138 | Bone-age deviation, per year | 2.38 (0.97 to 3.79) | 0.001 |
| Tanner stage I only \| height after bone-age adjustment | 138 | Boys vs girls | -1.04 (-3.40 to 1.31) | 0.383 |
| Tanner stage I only \| height after bone-age adjustment | 138 | BMI difference, per kg/m² | -0.54 (-0.99 to -0.09) | 0.019 |
| Tanner stage I only \| height before bone-age adjustment | 138 | Boys vs girls | -2.94 (-5.25 to -0.62) | 0.013 |
| Tanner stage I only \| height before bone-age adjustment | 138 | BMI difference, per kg/m² | -0.49 (-0.91 to -0.06) | 0.027 |
| Age <10 years \| bone-age deviation | 152 | Boys vs girls | -0.64 (-0.99 to -0.30) | <0.001 |
| Age <10 years \| bone-age deviation | 152 | BMI difference, per kg/m² | 0.03 (-0.03 to 0.08) | 0.348 |
| Age <10 years \| height after bone-age adjustment | 152 | Bone-age deviation, per year | 2.44 (1.14 to 3.73) | <0.001 |
| Age <10 years \| height after bone-age adjustment | 152 | Boys vs girls | -1.95 (-4.31 to 0.40) | 0.103 |
| Age <10 years \| height after bone-age adjustment | 152 | BMI difference, per kg/m² | -0.75 (-1.30 to -0.20) | 0.008 |
| Age <10 years \| height before bone-age adjustment | 152 | Boys vs girls | -3.52 (-6.03 to -1.01) | 0.006 |
| Age <10 years \| height before bone-age adjustment | 152 | BMI difference, per kg/m² | -0.69 (-1.23 to -0.15) | 0.012 |

**Note.** Values are unstandardized regression coefficients (β) with 95% confidence intervals estimated using HC3 heteroscedasticity-consistent standard errors. Sensitivity analyses included grouped Tanner adjustment (Tanner I, II–III, and IV–V), models without BMI adjustment, exclusion of observations with Cook's distance >4/N, and exclusion of observations below the 1st or above the 99th percentile of bone-age deviation. Subgroup analyses included Tibetan participants, participants with Tanner stage I, and participants aged <10 years. The Cook's distance threshold in the primary analytical sample was 4/199 = 0.0201. Subgroup analyses were not formal tests of between-subgroup heterogeneity. BMI, body mass index; CI, confidence interval; HC3, heteroscedasticity-consistent covariance estimator type 3. Models included sex, mean-centered chronological age and its squared term, ethnicity, BMI difference, and pubertal status (Tanner stage I vs >I); height models after bone-age adjustment additionally included bone-age deviation. Ethnicity was omitted in the Tibetan-only subgroup and pubertal status was omitted in the Tanner stage I subgroup because these variables were constant. Cook-influential observations were excluded if the threshold was exceeded in either the primary bone-age-deviation model or the height model after bone-age adjustment. Sample sizes were 199 for the primary, grouped-Tanner, and no-BMI analyses, 178 after influence exclusion, 195 after tail exclusion, 157 for Tibetan participants, 138 for Tanner stage I, and 152 for age <10 years. Bone-age deviation was calculated as bone age minus chronological age (years). Height difference and BMI difference were calculated as the measured value minus the corresponding age- and sex-specific national reference mean (cm and kg/m², respectively).

**Supplementary Table 4. Sex-related statistical indirect, direct, and total associations across sensitivity and subgroup analyses.**

| **Scenario** | **N** | **Association** | **Estimate** | **Percentile 95% CI** | **BCa 95% CI** |
| --- | --- | --- | --- | --- | --- |
| Primary | 199 | Indirect association | -1.78 | -2.89 to -0.83 | -3.02 to -0.90 |
| Primary | 199 | Direct association | -1.61 | -3.73 to 0.40 | -3.77 to 0.37 |
| Primary | 199 | Total association | -3.39 | -5.57 to -1.22 | -5.65 to -1.28 |
| Grouped Tanner sensitivity | 199 | Indirect association | -1.82 | -2.97 to -0.86 | -3.11 to -0.93 |
| Grouped Tanner sensitivity | 199 | Direct association | -1.53 | -3.66 to 0.48 | -3.70 to 0.45 |
| Grouped Tanner sensitivity | 199 | Total association | -3.35 | -5.61 to -1.20 | -5.66 to -1.25 |
| Cook-influential observations excluded | 178 | Indirect association | -0.90 | -1.63 to -0.32 | -1.71 to -0.36 |
| Cook-influential observations excluded | 178 | Direct association | -1.31 | -3.06 to 0.42 | -3.06 to 0.44 |
| Cook-influential observations excluded | 178 | Total association | -2.21 | -3.96 to -0.50 | -3.95 to -0.48 |
| Bone-age 1st/99th percentile tails excluded | 195 | Indirect association | -1.50 | -2.51 to -0.66 | -2.70 to -0.76 |
| Bone-age 1st/99th percentile tails excluded | 195 | Direct association | -1.65 | -3.68 to 0.43 | -3.78 to 0.34 |
| Bone-age 1st/99th percentile tails excluded | 195 | Total association | -3.15 | -5.21 to -1.03 | -5.42 to -1.18 |
| Tibetan participants only | 157 | Indirect association | -1.92 | -3.25 to -0.78 | -3.48 to -0.93 |
| Tibetan participants only | 157 | Direct association | -2.33 | -4.40 to -0.29 | -4.39 to -0.27 |
| Tibetan participants only | 157 | Total association | -4.25 | -6.38 to -2.20 | -6.45 to -2.26 |
| Tanner stage I only | 138 | Indirect association | -1.89 | -3.42 to -0.53 | -3.75 to -0.73 |
| Tanner stage I only | 138 | Direct association | -1.04 | -3.29 to 1.18 | -3.22 to 1.25 |
| Tanner stage I only | 138 | Total association | -2.94 | -5.19 to -0.74 | -5.16 to -0.72 |
| Age <10 years | 152 | Indirect association | -1.57 | -2.93 to -0.45 | -3.13 to -0.58 |
| Age <10 years | 152 | Direct association | -1.95 | -4.16 to 0.27 | -4.26 to 0.12 |
| Age <10 years | 152 | Total association | -3.52 | -5.85 to -1.25 | -6.14 to -1.44 |
| Age ≥10 years | 47 | Indirect association | NE | NE | NE |
| Age ≥10 years | 47 | Direct association | NE | NE | NE |
| Age ≥10 years | 47 | Total association | NE | NE | NE |

**Note.** Estimates represent differences in height difference, in centimeters, for boys relative to girls. Statistical indirect associations through bone-age deviation were estimated using the product-of-coefficients approach. Percentile and BCa 95% confidence intervals were obtained from 5,000 participant-level bootstrap resamples. Sensitivity analyses included grouped Tanner adjustment, exclusion of Cook-influential observations, and exclusion of observations below the 1st or above the 99th percentile of bone-age deviation. Subgroup analyses included Tibetan participants, Tanner stage I participants, and participants aged <10 or ≥10 years. Estimates for participants aged ≥10 years (n = 47) are reported as NE because unit leverage prevented reliable estimation under the specified modeling procedure. NE, not estimable; BCa, bias-corrected and accelerated; CI, confidence interval. These cross-sectional decompositions do not establish temporal or causal mediation. The primary adjustment set comprised mean-centered chronological age and its squared term, ethnicity, BMI difference, and Tanner stage I vs >I; grouped-Tanner models used stages I, II–III, and IV–V. Ethnicity was omitted in the Tibetan-only subgroup and pubertal status was omitted in the Tanner stage I subgroup because these variables were constant. Influence exclusion was based on Cook’s distance >4/199 in either the primary bone-age or bone-age-adjusted height model. N denotes the scenario-specific sample size. BMI, body mass index.

**Supplementary Table 5. Exploratory BMI-related statistical indirect, direct, and total associations through bone-age deviation.**

| **Scenario** | **N** | **Association** | **Estimate** | **Percentile 95% CI** | **BCa 95% CI** |
| --- | --- | --- | --- | --- | --- |
| Primary | 199 | Indirect association | 0.16 | 0.03 to 0.29 | 0.04 to 0.31 |
| Primary | 199 | Direct association | -0.50 | -0.91 to -0.08 | -1.01 to -0.14 |
| Primary | 199 | Total association | -0.34 | -0.76 to 0.08 | -0.84 to 0.03 |
| Grouped Tanner sensitivity | 199 | Indirect association | 0.16 | 0.04 to 0.29 | 0.06 to 0.32 |
| Grouped Tanner sensitivity | 199 | Direct association | -0.51 | -0.91 to -0.11 | -0.98 to -0.16 |
| Grouped Tanner sensitivity | 199 | Total association | -0.34 | -0.76 to 0.05 | -0.81 to 0.01 |
| Cook-influential observations excluded | 178 | Indirect association | 0.09 | 0.02 to 0.17 | 0.03 to 0.19 |
| Cook-influential observations excluded | 178 | Direct association | -0.18 | -0.47 to 0.14 | -0.47 to 0.13 |
| Cook-influential observations excluded | 178 | Total association | -0.09 | -0.38 to 0.23 | -0.39 to 0.22 |
| Bone-age 1st/99th percentile tails excluded | 195 | Indirect association | 0.16 | 0.05 to 0.29 | 0.07 to 0.33 |
| Bone-age 1st/99th percentile tails excluded | 195 | Direct association | -0.48 | -0.89 to -0.06 | -1.00 to -0.14 |
| Bone-age 1st/99th percentile tails excluded | 195 | Total association | -0.32 | -0.72 to 0.09 | -0.79 to 0.04 |

**Note.** Estimates represent differences in height difference, in centimeters, per 1-kg/m² increase in BMI difference from the age- and sex-specific national reference. Statistical indirect associations through bone-age deviation were estimated using the product-of-coefficients approach. Percentile and BCa 95% confidence intervals were obtained from 5,000 participant-level bootstrap resamples. Sensitivity analyses included grouped Tanner adjustment, exclusion of Cook-influential observations, and exclusion of observations below the 1st or above the 99th percentile of bone-age deviation. Because BMI mathematically incorporates height and the direct and indirect estimates may operate in opposite directions, these analyses are exploratory and should not be interpreted as conventional proportional mediation; no proportion mediated is reported. BMI, body mass index; BCa, bias-corrected and accelerated; CI, confidence interval. Models were adjusted for sex, mean-centered chronological age and its squared term, ethnicity, and Tanner stage I vs >I; grouped-Tanner models used stages I, II–III, and IV–V. The direct association was estimated after including bone-age deviation, and the total association before including it. Influence exclusion was based on Cook’s distance >4/199 in either the primary bone-age or bone-age-adjusted height model. N denotes the scenario-specific sample size. These cross-sectional estimates describe statistical associations and do not establish temporal ordering or causal mediation.

**Supplementary Table 6. Exploratory associations of endocrine and biochemical biomarkers with bone-age deviation and height difference.**

| **Model** | **N** | **Biomarker** | **Beta (95% CI)** | **P value** | **FDR q value** |
| --- | --- | --- | --- | --- | --- |
| Exploratory bone-age model | 199 | PTH, per pg/mL | 0.003 (-0.002 to 0.008) | 0.218 | 0.364 |
| Exploratory bone-age model | 199 | 25-hydroxyvitamin D, per ng/mL | 0.000 (-0.026 to 0.026) | 0.997 | 0.997 |
| Exploratory bone-age model | 199 | Calcium, per mmol/L | -0.176 (-1.235 to 0.883) | 0.744 | 0.826 |
| Exploratory bone-age model | 199 | Phosphorus, per mmol/L | 0.647 (-0.316 to 1.610) | 0.187 | 0.364 |
| Exploratory bone-age model | 199 | ALP, per U/L | 0.002 (-0.000 to 0.004) | 0.131 | 0.328 |
| Exploratory height model | 199 | PTH, per pg/mL | 0.009 (-0.031 to 0.048) | 0.665 | 0.826 |
| Exploratory height model | 199 | 25-hydroxyvitamin D, per ng/mL | -0.255 (-0.418 to -0.091) | 0.002 | 0.025 |
| Exploratory height model | 199 | Calcium, per mmol/L | 8.104 (-0.899 to 17.106) | 0.077 | 0.258 |
| Exploratory height model | 199 | Phosphorus, per mmol/L | 1.578 (-4.204 to 7.360) | 0.591 | 0.826 |
| Exploratory height model | 199 | ALP, per U/L | 0.018 (0.004 to 0.032) | 0.010 | 0.050 |

**Note.** Values are unstandardized regression coefficients (β) with HC3 heteroscedasticity-consistent 95% confidence intervals. PTH, 25-hydroxyvitamin D, calcium, phosphorus, and ALP were entered simultaneously into each model. The bone-age model was additionally adjusted for sex, centered chronological age, centered age squared, ethnicity, BMI difference, and grouped Tanner stage (I, II–III, and IV–V). The height-difference model additionally included bone-age deviation. Benjamini–Hochberg false discovery rate correction was applied across the 10 biomarker–outcome tests. These analyses were exploratory and hypothesis-generating. PTH, parathyroid hormone; ALP, alkaline phosphatase; BMI, body mass index; CI, confidence interval; FDR, false discovery rate; HC3, heteroscedasticity-consistent covariance estimator type 3. Bone-age deviation was calculated as bone age minus chronological age (years). Height difference and BMI difference were calculated as the measured value minus the corresponding age- and sex-specific national reference mean (cm and kg/m², respectively). Coefficients indicate the difference in bone-age deviation (years) or height difference (cm) per biomarker-unit increase specified in the table. All models included 199 participants. P values are unadjusted two-sided robust t-test P values; q values are Benjamini–Hochberg-adjusted P values and are displayed to three decimal places.

**Supplementary Table 7. Robust tests of sex-by-age and sex-by-BMI interactions for bone-age deviation and height difference.**

| **Outcome/model** | **Interaction tested** | **Robust F statistic** | **df1** | **df2** | **P value** |
| --- | --- | --- | --- | --- | --- |
| Bone-age deviation | Sex × age (linear + quadratic terms) | 0.666 | 2 | 189 | 0.515 |
| Height difference | Sex × age (linear + quadratic terms) | 0.327 | 2 | 188 | 0.721 |
| Bone-age deviation | Sex × BMI difference | 0.109 | 1 | 191 | 0.741 |

**Note.** All models included 199 participants. Interaction hypotheses were evaluated using type 3 heteroscedasticity-consistent (HC3) robust Wald F tests. The sex-by-age test jointly evaluates the sex × mean-centered age and sex × squared mean-centered age coefficients (2 numerator degrees of freedom). These models included sex, centered age and its squared term, ethnicity, BMI difference, and grouped Tanner stage (I, II–III, and IV–V); the height-difference model additionally included bone-age deviation. The sex-by-BMI-difference test evaluates one interaction coefficient (1 numerator degree of freedom) in the bone-age-deviation model adjusted for sex, centered age and its squared term, ethnicity, BMI difference, and Tanner stage I vs >I. df1 and df2 denote numerator and denominator degrees of freedom, respectively. P values are from the upper tail of the corresponding F distribution. BMI, body mass index.

**Supplementary Table 8. Regression model diagnostics.**

| **Model** | **N** | **Parameters** | **Residual df** | **Maximum leverage** | **Breusch-Pagan statistic** | **Breusch-Pagan df** | **Breusch-Pagan P value** | **Maximum Cook distance** |
| --- | --- | --- | --- | --- | --- | --- | --- | --- |
| Primary bone-age model | 199 | 7 | 192 | 0.1529 | 3.387 | 6 | 0.759 | 0.0905 |
| Height model before bone-age adjustment | 199 | 7 | 192 | 0.1529 | 21.686 | 6 | 0.001 | 0.3942 |
| Height model after bone-age adjustment | 199 | 8 | 191 | 0.1642 | 22.363 | 7 | 0.002 | 0.3804 |
| Bone-age model: grouped Tanner sensitivity | 199 | 8 | 191 | 0.1537 | 4.953 | 7 | 0.666 | 0.1269 |
| Height model: grouped Tanner sensitivity | 199 | 9 | 190 | 0.1642 | 23.279 | 8 | 0.003 | 0.3466 |
| Bone-age model: no BMI adjustment | 199 | 6 | 193 | 0.1526 | 3.429 | 5 | 0.634 | 0.1026 |
| Height model: no BMI adjustment | 199 | 7 | 192 | 0.1573 | 19.342 | 6 | 0.004 | 0.3727 |
| Bone-age model: sex by BMI interaction | 199 | 8 | 191 | 0.2479 | 3.564 | 7 | 0.828 | 0.0906 |
| Bone-age model: sex by age interaction | 199 | 10 | 189 | 0.2502 | 7.037 | 9 | 0.633 | 0.1460 |
| Height model: sex by age interaction | 199 | 11 | 188 | 0.2609 | 23.719 | 10 | 0.008 | 0.3234 |

**Note.** Model diagnostics include the number of model parameters, residual degrees of freedom, maximum leverage, Breusch–Pagan test statistic and P value, and maximum Cook's distance. The Breusch–Pagan test was used to evaluate heteroscedasticity. Because heteroscedasticity was present in some height models, HC3 heteroscedasticity-consistent standard errors were used for statistical inference in all principal models regardless of the Breusch–Pagan test result. Cook-influential observations used in the sensitivity analysis were defined using a threshold of 4/N (0.0201 for N = 199). BMI, body mass index; df, degrees of freedom; HC3, heteroscedasticity-consistent covariance estimator type 3. Parameter counts include the intercept. Maximum leverage and maximum Cook’s distance refer to the largest participant-level values within each fitted model. Influence exclusion was triggered by Cook’s distance exceeding the threshold in either the primary bone-age-deviation model or the height model after bone-age adjustment.
